# Bridging Perspectives: How Canadian Patients and Caregivers View Quality of Life in Multiple Myeloma Compared to Validated Instruments

**DOI:** 10.3390/curroncol33030174

**Published:** 2026-03-19

**Authors:** Julie Patenaude, Ariane Légaré, Florence Dupont, Gabriele Colasurdo, Martine Elias, Catherine Beauchemin, Jean Lachaine

**Affiliations:** 1 PeriPharm Inc., 420 Notre-Dame Ouest, Suite 501, Montreal, QC H2Y 1V3, Canada; julie.patenaude@peripharm.com (J.P.); ariane.legare@peripharm.com (A.L.); florence.dupont@peripharm.com (F.D.); catherine.beauchemin@peripharm.com (C.B.); 2 Myeloma Canada, 1255 TransCanada, Suite 160, Dorval, QC H9P 2V4, Canada; gcolasurdo@myeloma.ca (G.C.); melias@myeloma.ca (M.E.)

**Keywords:** multiple myeloma, patient-reported outcomes (PROs), caregiver-reported outcomes (CRO), quality of life (QoL), real-world evidence (RWE), patient perspective

## Abstract

**Background:** Validated patient- and caregiver-reported outcome (PRO and CRO) questionnaires are widely used to assess quality of life (QoL) in multiple myeloma (MM). However, most were developed before recent therapeutic advances that have modified disease trajectories and may no longer fully reflect patients’ and caregivers’ experiences. **Methods:** A Canadian cross-sectional observational study was conducted in collaboration with the PROxy Network and Myeloma Canada. Patients with MM completed the EORTC QLQ-C30, EORTC QLQ-MY20, EQ-5D-5L, and ESAS-R questionnaires and caregivers of patients with MM completed the CarGOQoL. Both groups rated their perceived QoL using a numerical rating scale. The strength of associations between validated questionnaire global scores and self-perceived QoL was assessed using Spearman’s correlation coefficient (r). **Results:** Between October 2024 and February 2025, the study enrolled 305 patients and 104 caregivers. Moderate correlations were observed between patients’ perceived QoL and validated PRO global scores (range of r: 0.59 to 0.65). The MM-specific QLQ-MY20 showed one of the lowest correlations (r = 0.59; 95% confidence interval (CI) 0.51–0.66; *p* < 0.001). The caregiver-reported CarGOQoL also demonstrated a moderate association (r = 0.54; 95% CI 0.38–0.67; *p* < 0.001). **Conclusions:** Contemporary treatment advances, including prolonged survival and evolving side effect profiles, may not be reflected in currently available PRO and CRO instruments.

## Simple Summary

Validated quality of life tools are commonly used to assess the impact of multiple myeloma on patients, and validated tools are also available to evaluate the quality of life of caregivers. However, most of these tools were created before recent treatment advances that have changed how people live with the disease. In this nationwide Canadian study, patients and caregivers of patients with multiple myeloma completed standard questionnaires and rated their own quality of life. Questionnaire scores were only moderately correlated with patients’ and caregivers’ self-perceived quality of life, indicating that meaningful dimensions of daily life and treatment burden may be insufficiently captured by current validated questionnaires. These findings indicate that evolving treatment contexts have altered lived experiences and underscore the need for updated multiple myeloma- and caregiver-specific quality-of-life instruments to ensure assessments remain relevant and meaningful.

## 1. Introduction

Multiple myeloma (MM) is the second most prevalent hematologic malignancy, accounting for approximately 1% of all cancers [1,2,3]. Over the past two decades, its incidence in Canada has slightly decreased in men by 0.4% per year and increased in women by 1.3% per year [4]. The disease predominantly affects older individuals, with a mean age of 70 years at diagnosis [3]. MM originates from malignant plasma cells found primarily in the bone marrow and typically evolves from an asymptomatic stage to a symptomatic phase characterized by pain, fatigue, weakness, neuropathy, and bone lesions [2,5,6,7]. As MM remains incurable in the majority of patients, current therapeutic strategies focus mainly on reducing the symptom burden, prolonging survival and delaying disease progression [8].

MM is associated with a substantial symptom burden and significantly poorer quality of life (QoL) compared to other hematologic cancers [9,10]. Consequently, the impact of the disease extends beyond patients to their families, with informal caregivers frequently taking on this role without adequate training or support systems [11]. Evidence suggests that caregivers may experience greater psychological distress and lower QoL than patients, with high rates of anxiety (44%) and symptoms of post-traumatic stress disorder (24%) frequently reported [11,12]. Moreover, advances in novel therapies have prolonged patient survival, thereby extending the duration of both the disease experience and caregiving responsibilities, ultimately contributing to increased caregiver burden [12,13]. Therefore, QoL remains a key consideration in MM care for both patients and caregivers [9,14].

Although several validated patient- and caregiver-reported outcome (PRO and CRO) questionnaires are widely used to assess QoL in MM, most were developed prior to the introduction of newer interventions and may not adequately capture patients’ current experiences and perspectives [15]. Supporting this concern, a recent survey revealed that patients with MM perceived that commonly used QoL instruments did not accurately capture their lived experiences when asked to evaluate the relevance of each item [15]. In parallel, systematic literature reviews have documented high heterogeneity in the design, analytical approaches, and findings of studies using these QoL tools [9,16]. Collectively, these observations underscore the need to enrich the content of MM-specific PROs and to develop higher-quality, more reliable instruments informed by the strengths and limitations of existing questionnaires [17]. This study therefore aimed to assess how QoL scores derived from commonly used and validated instruments correlate to self-reported QoL perceptions among adults with MM and caregivers of patients with MM.

## 2. Methods

### 2.1. Study Design and Population

A cross-sectional, observational study was conducted across Canada to capture a real-time assessment of patients with MM and caregivers of patients with MM. Eligible participants were adults (≥18 years) listed in the Myeloma Canada database as either patients with MM or current caregivers, and were able to read English or French. Recruitment was conducted via email invitation sent to all eligible individuals in the Myeloma Canada database, with a target sample size of 300 patients and 100 caregivers. The sample size was determined based on the precision of the confidence interval (CI), as wider CIs indicate greater uncertainty around the correlation estimates. For 300 patients, expected widths were 0.20 for correlation coefficient (r) = 0.30 (CI: 0.20–0.40) and 0.10 for r = 0.80 (CI: 0.75–0.85), while for 100 caregivers, expected CI widths were 0.40 for r = 0.30 (CI: 0.10–0.50) and 0.20 for r = 0.80 (CI: 0.70–0.90). The study had no exclusion criteria.

The study was approved by Veritas Independent Review Board, an independent ethics committee, and conducted in accordance with the principles of the Declaration of Helsinki. Recruitment took place between October 2024 and February 2025. All participants provided informed consent before enrollment and completed the survey either through the PROxy Network web-based platform (Qc, Canada) or, if preferred, on paper. Data confidentiality was ensured through encryption, the use of study identification codes, and restricted access to identifiable information. No protocol deviations were reported, and the study was registered on ClinicalTrials.gov (NCT06610045).

### 2.2. Data Collection

Data were collected at a single time point using self-administered questionnaires. Participants completed validated PRO and CRO instruments, a perceived QoL questionnaire, and a baseline form capturing demographic and disease-related information. Paper forms underwent double data entry, and all datasets were systematically reviewed for accuracy and potential outliers.

### 2.3. PRO Measures

For patients, QoL was assessed using several validated instruments designed to capture both general and disease-specific aspects of their experience with MM. The European Organisation for Research and Treatment of Cancer Quality of Life Questionnaire-Core 30 (QLQ-C30) is a validated, cancer-specific QoL questionnaire widely used in clinical trials [18]. It includes five functional scales (physical, role, emotional, cognitive and social functioning), three symptom scales (fatigue, pain, nausea, and vomiting), a global health status/QoL scale, and six single items assessing additional symptoms and financial impact, all based on a 7-day recall period. All scales and items are converted to a 0–100 score. Higher scores on functional and global health scales indicate better functioning and QoL, whereas higher scores on symptom scales reflect greater symptom burden [18].

The European Organisation for Research and Treatment of Cancer Quality of Life Questionnaire–Multiple Myeloma Module (QLQ-MY20) is administered alongside the QLQ-C30 to assess health-related QoL in patients with MM, also based on a 7-day recall period [19]. It comprises 20 items across four scales: two functional (future perspective and body image) and two symptom-related (disease symptoms and treatment side effects). Scores are linearly transformed to a 0–100 scale, with higher functional scores indicating better functioning and higher symptom scores reflecting greater symptom burden [19].

The EuroQol 5 Dimensions 5 Level (EQ-5D-5L) is a validated, generic instrument used to assess health-related QoL at the time of completion [20,21]. It includes five dimensions (mobility, self-care, usual activities, pain/discomfort, and anxiety/depression), each with five severity levels, allowing for 3125 possible health states. The EQ-5D-5L health states may be converted into a single summary index (utility value) using a country-specific value set. The Canadian value set was used in the present study, with scores ranging from −0.148 to 0.949 [22]. Scores usually range from 0 (a health state equivalent to death) to 1 (perfect health), with negative values possible to denote states perceived as worse than death [20,21].

The Edmonton Symptom Assessment System-Revised (ESAS-R) is a 10-item validated symptom scale used in Canadian cancer centres [23]. Patients rate 9 symptoms (e.g., pain, nausea, anxiety) on a 0–10 scale, with higher scores indicating greater severity according to how they feel at the time of questionnaire completion. A total distress score (range: 0–90) is calculated by summing each symptom, with a higher score indicating higher total symptom burden [24].

Finally, an in-house questionnaire was used to assess perceived QoL using two numeric rating scales (NRSs), each ranging from 1 (“best QoL you can imagine”) to 10 (“the worst QoL you can imagine”). One scale captured the impact of MM on QoL on the day of completion, while the other assessed the impact over the past week to align with the recall period of most of the validated questionnaires. A standardized definition of QoL was included in both questions to ensure consistent interpretation of QoL across participants. A free-text box was also provided for participants to describe in their own words how MM affected their QoL.

### 2.4. CRO Measures

The Caregiver Oncology Quality of Life questionnaire (CarGOQoL) is a validated 29-item questionnaire assessing caregiver QoL across 10 dimensions: psychological well-being, burden, relationship with healthcare, administration and finances, coping, physical well-being, self-esteem, leisure time, social support, and private life [25]. Each item is rated on a 5-point scale, reflecting experiences over the past 4 weeks. A summary index is derived from the dimension scores, with higher scores indicating better caregiver QoL [25].

In addition to the standardized instrument, caregivers were also invited to share their own perspective on QoL through an in-house questionnaire. They rated the impact of caring for a patient with MM on their QoL over the past 4 weeks using a NRS ranging from 1 (“best QoL you can imagine”) to 10 (“the worst QoL you can imagine”). To ensure consistent interpretation, the same standardized definition of QoL used in the patient questionnaire was provided. A free-text comment box allowed caregivers to elaborate in their own words how caregiving had affected their QoL.

### 2.5. Data Analysis

All analyses were conducted according to a pre-defined statistical analysis plan to ensure consistency and methodological rigor. Most data were captured electronically, while paper-based entries (2.69%) underwent double data entry.

Before conducting the statistical analyses, the completeness of the data was evaluated. Of 305 patients, 298 (97.7%) had complete datasets, and all caregiver records were complete. Missing data were handled per instrument-specific scoring guidelines. Out of the seven participants with missing items, one patient’s QLQ-C30 Total Score could not be calculated due to a missing single-item subscale (Financial Difficulties).

Descriptive and inferential statistical analyses were then performed to evaluate quantitative outcomes (questionnaires and NRS scores), while qualitative responses were analyzed (text box) using natural language processing (NLP) methods. Statistical analyses were conducted using R version 4.4.1 (New Zealand). Participant characteristics were summarized as frequencies and percentages for categorical variables, and as means (standard deviation [SD]) and medians (interquartile range [IQR]) for continuous variables. QoL results from the QLQ-C30, QLQ-MY20, EQ-5D-5L, ESAS-R, CarGOQoL, and NRS were reported per scale and total score. EQ-5D-5L utility values were derived using the Canadian value set, and ESAS-R symptom frequencies (score ≥ 1) and severity scores were presented [22]. Correlations between QoL global scores and perceived QoL (NRS) were assessed using only complete questionnaires, with recall periods matched. Shapiro–Wilk normality testing showed that only CarGOQoL was normally distributed. Therefore, Spearman’s correlation with bootstrap CIs was applied for all analyses. The correlation coefficient ranges from −1 to +1, with values closer to ±1 indicating stronger linear relationships and zero indicating no linear relationship. Interpretation was guided by commonly used thresholds and considered in the context of the analysis.

Free-text responses were analyzed separately to capture contextual nuances in participant experiences. These responses were processed using the Dcipher Analytics NLP platform (United Kingdom), which included translation of French entries, tokenization, lemmatization, removal of non-text elements, and part-of-speech tagging [26]. Topic modelling (correlation explanation) identified clusters of frequently co-occurring words, visualized as foam charts for patients and caregivers, and artificial intelligence (AI)-based theme detection highlighted key messages.

Finally, additional analyses were conducted to test the robustness of the findings and explore subgroup differences. Sensitivity analyses excluding outliers and adjusting for age, gender, presence of comorbidities, and time since diagnosis were conducted. Then, complementary analyses were performed to examine differences in correlations across subgroups. For patient QoL, analyses considered time since diagnosis (<1 year, ≥1–3 years, ≥3–5 years, ≥5 years), relapse status (relapse vs. no relapse), and the presence of a caregiver (caregiver vs. no caregiver). For caregiver QoL, correlations were evaluated according to relapse status (relapse vs. no relapse).

## 3. Results

### 3.1. Demographics and Disease Characteristics

A total of 305 patients with MM and 104 caregivers were recruited between October 2024 and February 2025. Patients showed an equal gender distribution (49.8% female and male), and most were aged between 60 and 79 years (75.4%). Most participants had been diagnosed either ≥1–3 years or ≥5 years prior to the study (63.6%). A majority had never experienced a relapse (67.5%), and most were currently receiving treatment (87.9%). Caregivers were predominantly female (74.0%) and were most often aged between 60–79 years (60.6%). Almost all were caring for a relative (98.1%). While 47.1% were retired, about 31.7% were employed full-time at the time of the survey. Table 1 presents patients’ and caregivers’ characteristics. Caregiver-reported patients’ characteristics can be found in Table S1.

### 3.2. PRO Results

The results of the QLQ-C30 revealed that the global health status of patients with MM was moderate, with a mean of 65.7 out of 100. All total scores are presented in Table 2 and detailed values are presented in Supplementary Materials. Overall, patients reported high levels of functioning across all scales, while experiencing few symptoms, with fatigue and insomnia being the most prominent. Detailed values for the functional and symptom scales of the QLQ-C30 are presented in Table S2. Similarly, findings from the QLQ-MY20 instrument revealed that patients with MM experienced a low level of symptomatology, consistent with their moderate functioning, with a total score of 70.3 All values for the functional and symptom scales of the QLQ-MY20 are provided in Table S3. In line with these results, the EQ-5D-5L indicated a moderate level of health with an overall EQ-5D index score of 0.80. The frequencies of reported problems and corresponding utility values of EQ-5D-5L are presented in Table S4, and the distribution of problem severity is illustrated in Figure S1. The ESAS-R confirmed that tiredness, impaired well-being, pain, and drowsiness were the most frequently reported symptoms; however, the mean total distress score (18.33) suggests an overall low symptom burden among patients. Detailed values for symptoms reported by patients of the ESAS-R are available in Table S5. Finally, patients rated their QoL as moderately positive, with a mean score of 6.6 for “today” and 6.5 for the “past week”, as indicated in Table 2. Ratings were consistent over time, suggesting stable perceived well-being despite disease-related challenges within a one-week period.

### 3.3. CRO Results

The results of CarGOQoL indicated a moderate caregiver QoL index over the past 4 weeks, with a mean score of 62.1, as reported in Table 3. This suggests that while caregivers are coping well overall, additional support is needed in certain areas. While caregivers reported high functioning in financial well-being, self-esteem, and burden, other domains such as psychological well-being, leisure time and private life and social support were notably lower. All index values of the CarGOQoL are presented in Table S6. Caregivers rated their QoL over the past four weeks with a mean NRS score of 6.1, reflecting a moderate self-perceived QoL while caring for an individual with MM, as indicated in Table 3.

## 4. Correlation Results

### 4.1. Patients

**Figure 1:**
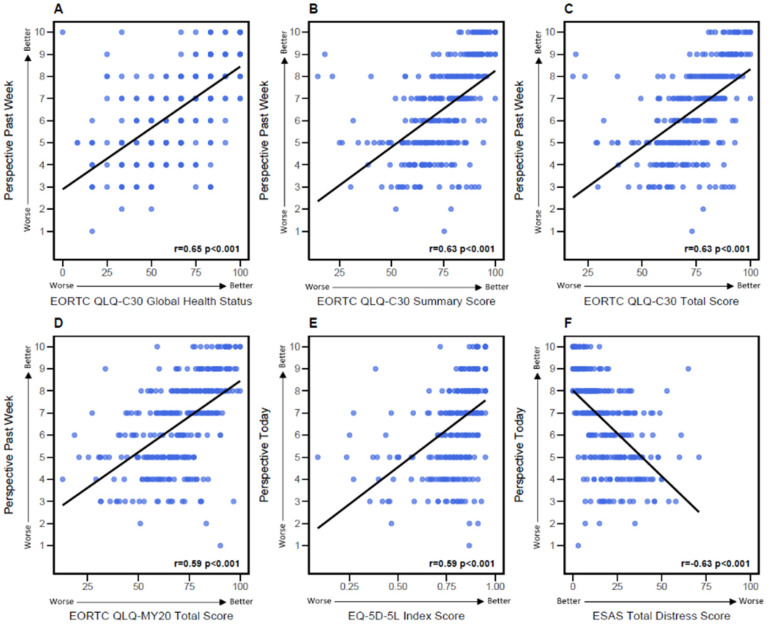
**Correlation between Patient Questionnaire Scores and Patient’s Perspective on QoL ^a^**. (**A**) EORTC QLQ-C30 Global Health Status Score and Patient’s Perspective. (**B**) EORTC QLQ-C30 Summary Score and Patient’s Perspective. (**C**) EORTC QLQ-C30 Total Score and Patient’s Perspective. (**D**) EORTC QLQ-MY20 Total Score and Patient’s Perspective. (**E**) EQ-5D-5L Index Score and Patient’s Perspective. (**F**) ESAS Total Distress Score and Patient’s Perspective. Blue dots represent individual patient scores, and the black line represents the correlation trend. Abbreviations: EORTC QLQ-C30 = European Organisation for Research and Treatment of Cancer Quality of Life questionnaire core-30 item; EORTC QLQ-MY20 = European Organisation for Research and Treatment of Cancer Quality of Life questionnaire Multiple Myeloma Module; EQ-5D-5L = EuroQol 5 Dimensions 5 Level; QoL = Quality of life; r = Correlation coefficient. ^a^ The correlation coefficient (r) reflects the linear relationship between the score and the patient’s perspective, ranging from −1 (perfect inverse correlation) to 1 (perfect positive correlation). A *p*-value < 0.05 indicates that r is significantly different than zero.

All PRO tools demonstrated a moderate correlation with patients’ own QoL ratings using the NRS. The QLQ-C30 Global Health Status showed the highest correlation (r = 0.65, *p* < 0.001), followed by the Summary and Total Scores (r = 0.63, *p* < 0.001), indicating consistent results across scoring methods for this questionnaire as illustrated in Figure 1A–C. The QLQ-MY20 also correlated moderately (r = 0.59, *p* < 0.001), suggesting that disease-specific QoL questionnaires do not necessarily provide stronger reflections of patients’ perceived QoL compared to non-specific questionnaires (Figure 1D). The EQ-5D-5L Index Score demonstrated a moderate correlation with NRS (r = 0.59, *p* < 0.001), while the ESAS-R Total Distress Score showed a moderate inverse correlation (r = −0.63, *p* < 0.001 (Figure 1E,F).

After adjusting for age, gender, presence of comorbidities, and time since diagnosis, all correlations weakened compared with the base case. Additional subgroup analyses revealed that correlations were weaker in patients diagnosed ≥1–3 years than in those diagnosed ≥3–5 years for the QLQ-C30 Summary Score, QLQ-C30 Total Score, and MY20 Total Score. For the same questionnaires, correlations were also weaker in patients with a caregiver compared with those without a caregiver, as well as in patients privately insured compared with those publicly insured. Both the EQ-5D-5L Index Score and the ESAS-R showed stronger correlations with the NRS in patients diagnosed ≥3–5 years, while the ESAS-R correlation was slightly weaker in patients who had relapsed. The scatter plots for sensitivity analyses for each global score are presented in Figures S2–S7 and Table S7.

### 4.2. Caregivers

**Figure 2:**
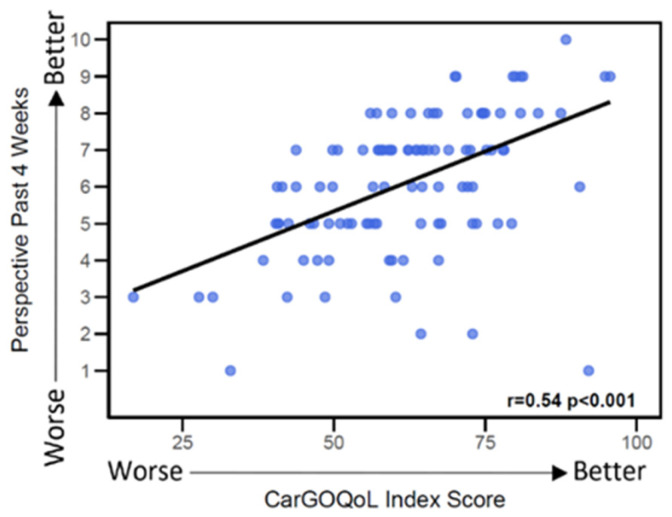
**Correlation between CarGOQoL Index Score and Caregiver’s Perspective on QoL ^a^**. Blue dots represent individual patient scores, and the black line represents the correlation trend. Abbreviations: CarGOQoL = CareGiver Oncology Quality of Life questionnaire; QoL = Quality of life; r = Correlation coefficient. ^a^ The correlation coefficient (r) reflects the linear relationship between the score and the patient’s “past 4 weeks” perspective, ranging from −1 (perfect inverse correlation) to 1 (perfect positive correlation). A *p*-value < 0.05 indicates that r is significantly different than zero.

A moderate positive correlation was observed between CarGOQoL Index score and caregivers’ NRS (r = 0.54; *p* < 0.001), as illustrated in Figure 2.

Sensitivity analysis controlling for age, gender, comorbidities and time since diagnosis was aligned with the base case scenario. However, the correlation was weaker in caregivers who had been caregiving ≥1–3 years compared to those caregiving for ≥5 years, and in patients who had been diagnosed for <1 year or ≥1–3 years compared with patients diagnosed ≥3–5 years or for ≥5 years. The correlation was not statistically significant for caregivers of patients diagnosed for <1 year. Scatter plots for sensitivity analyses for the CarGOQoL Index score are presented in Figure S8.

### 4.3. Qualitative Analysis Results

In addition to the quantitative analyses, qualitative findings provided valuable context to better understand the lived experience of patients and caregivers. Patients described loss of autonomy, isolation resulting from immunosuppression, and emotional stress as key factors influencing their QoL (Table 4). Caregivers reported emotional and physical strain, isolation and financial burdens, dimensions that are partially represented by the CarGOQoL, which inadequately reflects the disruption of daily routine, personal roles and meaningful activities (Table 5).

## 5. Discussion

Overall, the assessment of QoL among patients with MM revealed relatively good functioning and a nuanced QoL profile. The QLQ-C30 global health score, EQ-5D-5L index value, and NRS ratings for “today” and for “the past week” suggests that patients generally perceive their QoL as relatively preserved. This finding is consistent with the index values of the general population in Canada (ranging from −0.15 to 0.95) [22]. The consistency across instruments indicates that generic and disease-specific measures capture similar trends in overall well-being. However, the lower social and functioning scores highlight an ongoing psychosocial burden that may persist even when symptoms are well managed. This disconnect between physical and psychological domains suggests that standard QoL instruments may underrepresent social isolation, emotional distress, and loss of autonomy, as echoed in patients’ qualitative comments. Notably, while fatigue, insomnia, pain and diarrhea were less frequently reported in the ESAS-R, these symptoms remained among the most influential on daily life, as per the QLQ-C30. This reinforces that mild but persistent symptoms can meaningfully affect patients’ perceived well-being. While fatigue is most common with the QLQ-C30, this symptom is not captured by all tools. Furthermore, qualitative findings from patients highlighted aspects that the EQ-5D, QLQ-C30 and QLQ-MY20 instruments failed to capture, such as the impact of transplantation, financial anxiety and care-related burden.

Despite these generally positive perceptions, existing PRO instruments, including the QLQ-MY20, developed specifically for MM, showed only moderate correlation with patients self-perceived QoL. This observation aligns with findings from a recent survey indicating that commonly used questionnaires (e.g., QLQ-C30, QLQ-MY20, EQ-5D-5L) do not adequately reflect the lived realities of patients with MM, as participants questioned the relevance of several items [15]. One potential explanation is that these questionnaires were developed when treatment regimens, survival expectations, and patient experiences differed substantially from today. Accordingly, advances in treatment and improved survival in multiple myeloma have altered the patient experience, with patients now spending less time with active disease and more time in remission while receiving ongoing therapy, during which they may experience a distinct set of treatment-related symptoms, such as fatigue, weakness, and psychological burden. As a result, several key modern domains expressed by patients are not adequately addressed. These include the challenges of living with immunosuppression, which significantly affects daily life and social interaction, and according to patients’ comments the physical and emotional burden associated with transplantation and the logistical stress from managing frequent appointments and complex medication schedules [27].

A similar pattern emerged among caregivers. The CarGOQoL, originally designed to measure the QoL of caregivers of patients with cancer, demonstrated only moderate correlation with caregivers’ own perspectives. Correlation with the CarGOQoL score appeared to be stronger among caregivers of patients who had not experienced a relapse, suggesting that the instrument may be more sensitive to the experiences of those supporting patients in a stable phase of the disease. Moreover, caregivers of patients diagnosed long ago also showed better alignment between CarGOQoL scores and perceived QoL, although the small size of this subgroup limits the confidence of this observation.

Qualitative insights from caregivers further underscore the limitations of the CarGOQoL in fully capturing the caregiver experience in the MM context. Caregivers described several aspects of their experience that are not well reflected in existing instruments. They emphasized the emotional burden associated with complex care logistics, financial anxiety, and feelings of isolation or loss of social connection, which are only partially encompassed within broader domains such as burden or leisure time. Moreover, the impact of caregiving on daily routines, personal roles, and engagement in meaningful personal or professional activities highlighted by patients also seems insufficiently captured by current tools. Given the critical role of caregivers in the MM care continuum and the substantial proportion of patients who depend on them, there is a strong rationale for developing a caregiver-specific CRO tool or module tailored to the unique challenges of providing care in MM.

To our knowledge, this study is among the few studies conducted in Canada to assess the correlation between QoL scores and patients’ and caregivers’ perceived quality of life in MM. Although burden has been widely recognized as a determinant of QoL for both patients and caregivers, evidence exploring its correlation with perceived QoL remains limited [28,29]. Evidence on the correlation between patient- and caregiver-reported QoL outcomes in other disease areas also remains scarce, limiting direct comparisons with our findings. While previous qualitative studies have explored the lived experiences and priorities of patients with MM, our study extends this literature by quantitatively linking patient- and caregiver-reported perceptions of QoL with validated tools [30].

Accordingly, there is a clear need for the development of an updated MM-specific PRO instrument that more accurately reflects the lived experiences and needs of this population. In support of this, the European Organisation for Research and Treatment of Cancer is currently revising the QLQ-MY20 to improve its sensitivity to evolving treatment contexts and patient experiences [19]. In the adjusted analysis of the current study, weaker correlations were observed after accounting for age, gender, and comorbidities, suggesting these factors may influence perceived QoL.

This study presents several notable strengths that enhance the credibility and relevance of its findings. First, the inclusion of more than 300 patients provided a robust sample size, thereby strengthening the reliability and precision of the results. The use of multiple validated PRO instruments, directly compared with patients perceived QoL, allowed for a comprehensive assessment of how standardized tools align with lived experiences. Another important strength lies in the inclusion of caregiver perspectives, which were assessed using a validated CRO tool. This dual perspective captures both sides of MM experience, enriching the understanding of how the disease affects not only patients but also those who support them. Data completeness was high across all instruments, supporting the reliability of the overall findings. Only limited missing responses were identified, including one case in which the QLQ-C30 Total Score could not be computed due to a missing Financial Difficulties item. This minor omission is not expected to have affected the robustness or interpretability of the results. Furthermore, the application of NLP to analyze free-text responses added qualitative depth to the quantitative findings, enabling the identification of additional patient- and caregiver-reported themes not captured by the standardized questionnaires. Additionally, subgroup and sensitivity analyses confirmed the robustness of the base-case findings, reinforcing confidence in the consistency of the observed correlations. Finally, to assess the robustness of our findings, we examined the correlations between the validated HRQoL instruments. Spearman correlation coefficients were 0.67 (0.60–0.73) between the QLQ-C30 global health score and the EQ-5D index, and 0.66 (0.59–0.72) between the QLQ-MY20 total score and the EQ-5D index. These results indicate moderate correlations, consistent with a previously published mapping analysis in patients with MM that reported a correlation of 0.70 between QLQ-C30/QLQ-MY20 scores and EQ-5D utility values [31].

Some limitations should nevertheless be acknowledged when interpreting these results. A potential selection bias cannot be excluded, as study participants were generally healthier and more educated than the broader MM population [32]. This pattern likely reflects the recruitment method used in this study. In addition, the use of an NRS to capture subjective QoL perception may have contributed to clustering toward the midpoint, reflecting a common tendency among respondents to avoid extreme values [33]. Similarly, the inclusion of participants with less symptomatic disease at the time of the study may have biased the overall rating toward a more moderate impact of MM on QoL. Nevertheless, this potential bias is expected to be consistent across both validated instruments and the in-house instrument, as all questionnaires were completed at the same timepoint. Moreover, eligibility based on self-reporting introduces a potential for misclassification or inconsistencies in diagnosis status. The interpretation of correlation results relied on the range coefficient, which is inherently arbitrary but commonly applied in the literature to contextualize the strength of associations [34]. In addition, given the cross-sectional study design, data were collected at a single time point, while MM is a heterogeneous and evolving disease. Longitudinal assessments would provide valuable insight into how QoL changes across different stages of the disease and treatment course. Finally, comorbidities were self-reported using a predefined checklist and were not clinically validated. Consequently, conditions such as anemia or kidney disease may represent either independent comorbidities or manifestations of MM or its treatment. Future studies with clinically validated data may help distinguish disease-related events from independent comorbidities. Despite these limitations, the results provide meaningful insights into the correlation between validated PRO and CRO instruments and the lived experiences of patients with MM and their caregivers.

## 6. Conclusions

The findings of this study underscore the importance of updating and refining PROs and CROs instruments to better reflect the lived experiences of both patients and caregivers. By comparing validated QoL tools with perceived QoL ratings, this study highlights important gaps in the ability of existing instruments to capture key aspects of life with MM and the burden of caregiving. These results emphasize the need for continued development of MM-specific and caregiver-specific measures that are sensitive to current treatment contexts, patient expectations, and evolving care dynamics, thereby ensuring that QoL assessments remain relevant within the evolving MM treatment landscape.

## Figures and Tables

**Table 1:** Baseline and Disease Characteristics of the Included Population.

Characteristics	Patients N = 305	Caregivers N = 104
**Gender, *n* (%)**		
Female	152 (49.4)	77 (74.0)
Male	152 (49.8)	27 (26.0)
Prefer not to answer	1 (0.3)	0 (0)
**Age, mean (SD)**	65.6 (9.0)	61.61 (12.4)
**Age, *n* (%)**		
<40 years	3 (0.98)	5 (4.8)
40–49 years	17 (5.6)	13 (12.5)
50–59 years	47 (15.4)	20 (19.2)
60–69 years	123 (40.3)	39 (37.5)
70–79 years	107 (35.1)	24 (23.1)
≥80 years	8 (2.6)	3 (2.9)
**Highest Education Level, *n* (%)**		
Elementary	3 (0.98)	1 (0.96)
High School	51 (16.7)	15 (14.4)
College	60 (19.7)	38 (36.5)
University	191 (62.6)	50 (48.1)
**Employment Status, *n* (%)**		
Employed full-time	39 (12.8)	33 (31.7)
Employed part-time	13 (4.3)	5 (4.8)
Retired	195 (63.9)	49 (47.1)
Unemployed	2 (0.66)	5 (4.8)
Self-employed	15 (4.9)	9 (8.7)
Invalid/Sick Leave	41 (13.4)	3 (13.4)
**Type of Medical Insurance, *n* (%)**		
Private	127 (41.6)	-
Public	75 (24.6)	-
Private and Public	96 (31.5)	-
Unknown	7 (2.3)	-
**Province of Residence, *n* (%)**		
British Columbia	49 (16.1)	16 (15.4)
Alberta	30 (9.8)	9 (8.7)
Saskatchewan	9 (3.0)	5 (4.8)
Manitoba	9 (3.0)	5 (4.8)
Ontario	121 (39.7)	43 (41.4)
Quebec	68 (22.3)	19 (18.3)
**Other provinces & territories ^a^**	19 (6.2)	6 (6.7)
**Comorbidities, *n* (%)**		
No	107 (35.1)	-
Unknown	3 (0.98)	-
Yes ^b^	195 (63.9)	-
** High Blood Pressure**	69 (34.8)	-
** Cancer**	45 (22.7)	-
** Osteoarthritis, Degenerative Arthritis**	41 (20.7	-
** Heart disease**	36 (18.2)	-
** Anxiety**	29 (14.6)	-
** Depression**	27 (13.6)	-
** Kidney disease**	25 (12.6)	-
** Diabetes**	24 (12.1)	-
** Anemia**	19 (9.6)	-
** Lung disease**	15 (7.6)	-
** Stomach disease**	11 (5.6)	-
** Other ^c^**	42 (21.5)	-
**Number of years since diagnosis, *n* (%)**		
<1 year	42 (13.8)	-
Between ≥1 and <3 years	101 (33.1)	-
Between ≥3 and <5 years	69 (22.6)	-
≥5 years	93 (30.5)	-
**Myeloma Setting, *n* (%)**		
Relapse	83 (27.2)	-
No relapse	206 (67.5)	-
Unknown	16 (5.3)	-
**Currently treated for myeloma, *n* (%)**	268 (87.9)	-
**Presence of a Caregiver, *n* (%)**	240 (78.7)	-
**Number of years since being a caregiver, *n* (%)**		
<1 year	-	24 (23.1)
Between ≥1 and <3 years	-	33 (31.7)
Between ≥3 and <5 years	-	11 (10.6)
≥5 years	-	36 (34.6)
**Caregiver Relationship with Patient, *n* (%)**		
Partner/spouse/family member	-	102 (98.1)
Friend/neighbor	-	2 (1.9)

Abbreviation: SD = Standard deviation. ^a^: New Brunswick: 5 (1.6%), Nova Scotia: 9 (3.0%), Prince Edward Island: 3 (0.98%), Newfoundland and Labrador: 2 (0.66%), and Territories: 0. ^b^: Participant could select more than one option. ^c^: Liver disease: 2 (1.0%), Rheumatoid arthritis: 7 (3.5%), Neuropathy: 4 (2.0%), Bone pain/disease: 4 (2.0%), Amyloidosis: 3 (1.5%), Glaucoma: 2 (1.0%), Sleep apnea: 2 (1.0%), Thyroid: 2 (1.0%), Autoimmune disease: 2 (1.0%), Ménière syndrome: 1 (0.5%), and Other: 13 (6.6%).

**Table 2:** Patients’ QoL Score Values.

	Scores N = 305
QoL Assessed by Validated Questionnaires	
**EORTC QLQ-C30 ^a^**	
Global health status, mean (SD)	65.7 (22.1)
Summary score, mean (SD)	75.1 (15.6)
Total score, mean (SD)	75.0 (15.5)
**EORTC QLQ-MY20 ^b^**	
Total score, mean (SD)	70.3 (17.2)
**EQ-5D-5L ^c^**	
Index value, mean (SD)	0.80 (0.14)
**ESAS-R ^d^**	
Total distress score, mean (SD)	18.3 (14.0)
**Patients’ Perspective ^e^**	
**Today**	
Mean (SD)	6.6 (2.0)
Median (IQR)	7 (5–8)
**Past week**	
Mean (SD)	6.5 (2.0)
Median (IQR)	7 (5–8)

Abbreviations: EORTC QLQ-C30 = European Organisation for Research and Treatment of Cancer Quality of Life. Questionnaire-Core 30; EORTC QLQ-MY20 = European Organisation for Research and Treatment of Cancer Quality of Life Questionnaire–Multiple Myeloma Module; EQ-5D-5L = EuroQol 5 Dimensions 5 Level; ESAS-R = Edmonton Symptom Assessment System-Revised; IQR = Interquartile Range (25th–75th percentile); QoL = Quality of life; SD = Standard deviation. ^a^ Scores range from 0 to 100. Higher scores indicate better functioning/QoL (functional scales and summary/total scores) or more severe symptoms (symptom scales). ^b^ Scores range from 0 to 100. Higher scores indicate better functioning/QoL (functional scales and total score) or more severe symptoms (symptom scales). ^c^ Index scores range from 0 to 1, where 0 is the value of a health state equivalent to being dead; negative values represent values as worse than death, and a value of 1 represents the value of full health. ^d^ The symptom scores range from 0 to 10 where 0 represents absence of the symptom and 10 represents the worst possible severity. The ESAS total distress score is derived from the sum of patient responses to each symptom, excluding “Other”, and thus ranges from 0 to 90. ^e^ Patients’ perspective scores range from 1 to 10. Higher scores indicate a better perspective on QoL.

**Table 3:** Caregivers’ QoL Score Values.

	Caregivers N = 104
QoL Assessed by Validated Questionnaires	
**CarGOQoL ^a^**	
CarGOQoL Index, mean (SD)	62.1 (15.1)
**Caregivers’ Perspective ^b^**	
**Past 4 weeks**	
Mean (SD)	6.1 (1.9)
Median (IQR)	6 (5–7)

Abbreviations: CarGOQoL = CareGiver Oncology Quality of Life questionnaire; IQR = Interquartile Range (25th–75th percentile); QoL = Quality of life; SD = Standard deviation. ^a^ CarGOQoL scores range from 0 to 100. Higher scores indicate a better QoL. ^b^ Caregivers’ perspective scores range from 1 to 10. Higher scores indicate a better perspective on QoL.

**Table 4:** Impact of Living with Myeloma.

Theme	Quote
Loss of autonomy	“Pain is excruciating making hard to sleep or get comfortable anywhere. I have trouble finding words. I am losing strength in my hands which makes doing anything extremely hard. I feel guilty not being able to do things… or having my wife do more.”—P1 “I am unable to do the regular things prior to myeloma like taking long walks at a good pace, cleaning my apartment without having to sit down and rest very often, having shortness of breath after I get out of the shower for example and I would say the worst is the lack of sleep at night and having to take a nap during the day.”—P2
Social Isolation Due to Immunosuppression	“No large group activities i.e. indoor sporting events, concerts, church (because of low immunity I don’t participate in anything that involves large groups). We go to restaurants at the least busy times to avoid crowds and always sit in a booth.”—P3 “I have very low immunity because of my current Multiple Myeloma treatment, therefore I am not able to join many social activities. A year ago I spent a month in the hospital with Influenza A and life-threatening pneumonia, which I had trouble fighting due to my low immunity. My lungs are still badly scarred, and I am very concerned that if I contract a virus that includes lung disease again, my lungs will not be able to get through it. My closest family members include children, teachers, and a nursing home worker. I do not see them in person over the winter unless it is outdoors at a distance.”—P4
Emotional Stress	“Fear and worry of dying young and not being able to raise my child. Being a burden on my family financially, emotionally and physically. Lack of enjoyment from life, such as travelling, playing sports. Constant fear of falling ill during travel and injuries from participating in sports or recreational activities.”—P5 “A feeling of Anxiety with what my life might become.”—P6

**Table 5:** Impact of Caregiving for a Patient with Myeloma.

Theme	Quote
Caregiver Burden and Impact on Daily Life	“Being very tired and experiencing role conflict with school, work, and caregiving. Leaves little time for myself or other tasks related to myself. It is difficult for me to connect with others my age due to being the first person in my friend group to experience caregiving.”—C1 “Feeling guilty and limiting my time doing things on my own or with a friend. Depression is setting in, and I feel down about myself and not keeping up with my physical health ie exercising and healthy foods. Too many thoughts about the “what ifs” and being alone eventually in the future.”—C2
Emotional and Physical Strain	“Caring for my mom has been extremely difficult, mentally more than physically. The thought that one day she will succumb to her disease is a thought that enters my mind multiple times a week and is very exhausting. I will sometimes hear about individuals getting married or having kids, and I have an urge to cry because I am scared my mom will not be there to experience those things with me. It is painful to watch the stress it has put on my father physically and mentally, and watch him develop stress-induced cardiac events as a result of it.”—C3 “It is stressful knowing that at any time it could turn for the worse, and as much as we can do to help him feel comfortable, there’s really nothing we can do. its stressful to not know what is going through his mind, because as much as it is hard for us I can’t imagine the stress of that knowledge on him.”—C4
Social Isolation	“My wife is post ASCT and has been in the maintenance phase for 5.5 years and the only significant impact is in our risk assessment of indoor group activities. We do not participate in activities where we feel our ability to be comfortable with our/her vulnerabilities to viral infection is questionable due to such things as quantity of people, size of facility, air flows, etc.”—C5 “Unable to attend any Christmas concerts, craft fairs or gatherings (other than immediate family) because of the constant fear of contracting an infection of some kind. Even attending such events on my own doesn’t seem wise as I might bring an infection home.”—C6

## Data Availability

The original contributions presented in the study are included in the article/Supplementary Materials; further inquiries can be directed to the corresponding author.

## Supplementary Materials

The following supporting information can be downloaded at: https://www.mdpi.com/article/10.3390/curroncol33030174/s1, Table S1: Caregiver-Reported Patient Characteristics; Table S2: EORTC QLQ-C30 – Score Values; Table S3: EORTC QLQ-MY20 – Score Values; Table S4: EQ-5D-5L Frequency of Reported Problems and Utility Values; Figure S1: Distribution of the EQ-5D-5L Problem Severity; Table S5: ESAS-R Symptoms Experienced by Patients; Table S6: CarGOQoL Index Values; Figure S2: Scatter Plots for EORTC QLQ-C30 Global Health Status and Patient’s Perspective on QoL; Figure S3: Scatter Plots for EORTC QLQ-C30 Summary Score and Patient’s Perspective on QoL; Figure S4: Scatter Plots for EORTC QLQ-C30 Total Score and Patient’s Perspective on QoL; Figure S5: Scatter Plots for EORTC QLQ-MY20 Total Score and Patient’s Perspective on QoL; Figure S6: Scatter Plots for EQ-5D-5L Index Score and Patient’s Perspective on QoL; Figure S7: Scatter Plots for ESAS-R Total Distress Score and Patient’s Perspective on QoL; Figure S8: Scatter Plots for CarGOQoL Index Score and Caregiver’s Perspective on QoL. Table S7: Questionnaire correlation with patient’s perspective—Subgroups analysis by type of insurance.
