# Supplementary material for: Bridging Perspectives: How Canadian Patients and Caregivers View Quality of Life in Multiple Myeloma Compared to Validated Instruments
Source: Curr Oncol. 2026 Mar 19;33(3):174. doi: 10.3390/curroncol33030174 (PMC13025678; doi:10.3390/curroncol33030174)
Supplement: Supplementary file 1 [file curroncol-33-00174-s001.zip › curroncol-4179649-supplementary.pdf]

Supplementary

Table S1. Caregiver-Reported Patient Characteristics

Characteristics	Caregivers' Patients N = 104
**Gender, n (%)**	
Female	33 (31.73)
Male	71 (68.27)
**Age, mean (SD)**	64.73 (11.35)
**Age, n (%)**	
< 40 years	3 (2.88)
40-49 years	6 (5.77)
50-59 years	25 (24.04)
60-69 years	33 (31.73)
70-79 years	27 (25.96)
≥80 years	10 (9.62)
**Comorbidities, n (%)**	
No	33 (31.73)
Unknown	2 (1.92)
Yes	69 (66.35)
High Blood Pressure	24 (23.08)
Cancer	20 (19.23)
Osteoarthritis, Degenerative Arthritis	16 (15.38)
Heart Disease	12 (11.54)
Anemia or Other Blood Disease	11 (10.58)
Anxiety	10 (9.62)
Diabetes	10 (9.62)
Kidney Disease	9 (8.65)
Other	31 (29.81)
**Number of years since diagnosis, n (%)**	
<1 year	27 (25.96)
Between ≥1 and <3 years	30 (28.85)
Between ≥3 and <5 years	10 (9.62)
≥5 years	37 (35.58)
**Myeloma Setting, n (%)**	
Relapse	34 (32.69)
No relapse	64 (61.54)
Unknown	6 (5.77)
**Currently treated for myeloma, n (%)**	94 (90.38)

Abbreviation: SD = Standard deviation.

Copyright© PeriPharm Inc. Confidential

Table S2. EORTC QLQ-C30 - Score Values [a]

	Mean (SD)	Median (IQR)
	N =305	
**Global Health Status**	65.68 (22.07)	66.67 (50.00 - 83.33)
**Functional Scales**		
Physical Functioning	78.82 (20.84)	86.67 (66.67 - 93.33)
Role Functioning	70.93 (27.61)	66.67 (66.67 - 100.00)
Emotional Functioning	75.90 (20.62)	75.00 (66.67 - 91.67)
Cognitive Functioning	75.46 (20.99)	83.33 (66.67 - 83.33)
Social Functioning	66.72 (27.89)	66.67 (50.00 - 83.33)
**Symptom Scales**		
Fatigue	37.81 (23.72)	33.33 (22.22 - 55.56)
Nausea and Vomiting	8.36 (17.84)	0.00 (0.00 - 16.67)
Pain	27.16 (25.71)	16.67 (0.00 - 50.00)
Dyspnea	22.62 (26.25)	33.33 (0.00 - 33.33)
Insomnia	36.72 (30.34)	33.33 (0.00 - 66.67)
Appetite Loss	12.90 (23.44)	0.00 (0.00 - 33.33)
Constipation	17.92 (26.75)	0.00 (0.00 - 33.33)
Diarrhea	27.65 (31.13)	33.33 (0.00 - 33.33)
Financial Difficulties[b]	18.97 (27.52)	0.00 (0.00 - 33.33)
**Summary Score**	75.13 (15.56)	78.03 (65.73 - 86.37)

Abbreviation: **EORTC QLQ-C30** = European Organization for Research and Treatment of Cancer Quality of Life questionnaire core-30 item; **IQR** = Interquartile Range (25th–75th percentile); **SD** = Standard deviation

[a] Scores range from 0 to 100. Higher scores indicate better functioning /QoL (functional scales and summary/total scores) or more severe symptoms (symptom scales).

[b] One participant selected two responses for the financial difficulties item. As a result, the item was marked as missing for this participant. Since it is a single-item scale, the Total Score could not be calculated for this participant.

Table S3. EORTC QLQ-MY20: Score Values [a]

	Mean (SD)	Median (IQR)
	N =305	
**Functional Scales**		
Body Image	70.93 (32.56)	66.67 (66.67 - 100.00)
Future Perspective	56.03 (25.49)	55.56 (44.44 - 77.78)
**Symptom Scales**		
Disease Symptoms	23.22 (18.58)	22.22 (11.11 - 33.33)
Side Effects of Treatment	22.36 (14.45)	22.22 (11.11 - 33.33)

Abbreviations: **EORTC QLQ-MY20** = European Organization for Research and Treatment of Cancer Quality of Life questionnaire Multiple Myeloma Module; **IQR** = Interquartile Range (25th–75th percentile); **SD** = Standard deviation.

[a] Scores range from 0 to 100. Higher scores indicate better functioning/QoL (functional scales and total score) or more severe symptoms (symptom scales).

Copyright© PeriPharm Inc.

Table S4. EQ-5D-5L Frequency of Reported Problems and Utility Value [a]

	Mobility n (%)	Self-Care n (%)	Usual Activities n (%) N =305	Pain / Discomfort n (%)	Anxiety / Depression n (%)
No problems	181 (59.34)	267 (87.54)	119 (39.02)	66 (21.64)	127 (41.64)
Slight problems	72 (23.61)	32 (10.49)	117 (38.36)	152 (49.84)	129 (42.30)
Moderate problems	42 (13.77)	6 (1.97)	51 (16.72)	76 (24.92)	37 (12.13)
Severe problems	9 (2.95)	0	14 (4.59)	8 (2.62)	11 (3.61)
Extreme problems / Unable to do	1 (0.33)	0	4 (1.31)	3 (0.98)	1 (0.33)

Abbreviations: **EQ-5D-5L** = EuroQol 5 Dimensions 5 Level; **IQR** = Interquartile Range (25th–75th percentile) ; **SD** = Standard deviation
[a]Index scores range from 0 to 1, where 0 is the value of a health state equivalent to being dead; negative values represent values as worse than death, and a value of 1 represents the value of full health.

Figure S1. Distribution of the EQ-5D-5L Problem Severity [a]

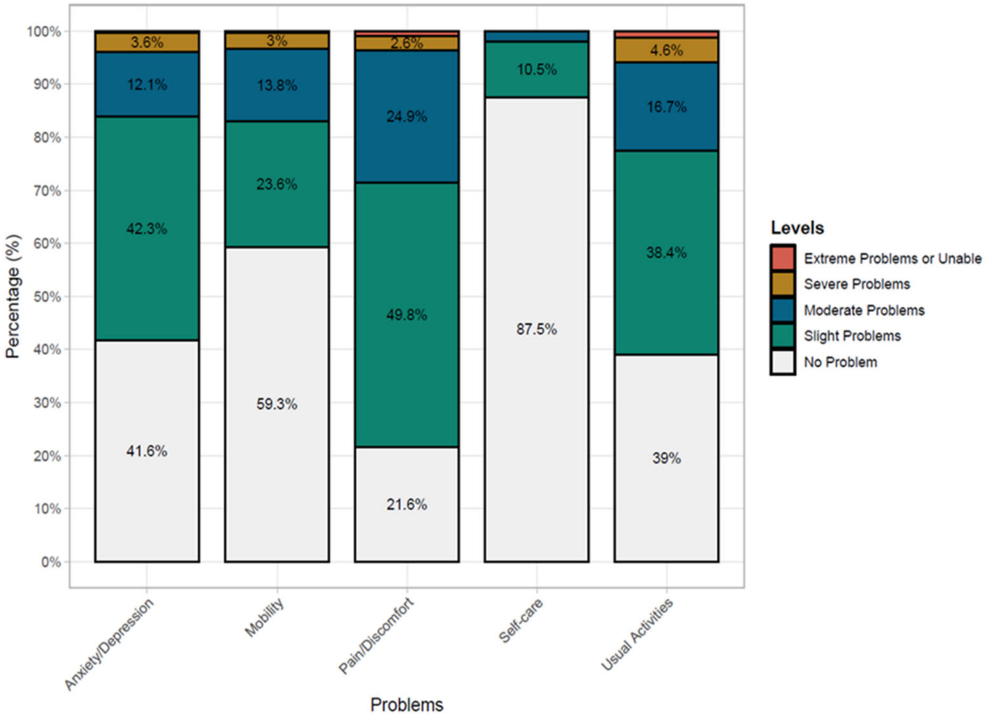

Abbreviations: **EQ-5D-5L** = EuroQol 5 Dimensions 5 Level
[a] Percentage <2% are not presented in the graph.

Table S5. ESAS-R Symptoms Experienced by Patients [a]

	n (%)	Score, Mean (SD) N =305	Score, Median (IQR)
**Pain**	234 (76.73)	2.32 (2.14)	2 (1 – 4)
**Tiredness**	274 (89.84)	3.49 (2.46)	3 (2 – 5)
**Drowsiness**	211(69.18)	2.50 (2.49)	2 (0 – 4)
**Nausea**	48 (15.74)	0.44 (1.39)	0 (0 – 0)
**Lack of Appetite**	110 (36.07)	1.19 (2.06)	0 (0 – 2)
**Shortness of Breath**	156 (51.15)	1.52 (2.12)	1 (0 – 2)
**Depression**	164 (53.77)	1.77 (2.36)	1 (0 – 3)
**Anxiety**	190 (62.30)	2.08 (2.46)	1 (0 – 3)
**Well-being**	264 (86.56)	3.02 (2.20)	3 (1 – 5)
**Other**	160 (52.46)	2.71 (3.11)	1 (0 – 5)

Abbreviation: **ESAS-R** = Edmonton Symptoms Assessment Scale – Revised; **IQR** = Interquartile Range (25th–75th percentile); **SD** = Standard deviation.

[a]The symptom scores range from 0 to 10 where 0 represents absence of the symptom and 10 represents the worst possible severity. The ESAS total distress score is derived from the sum of patient responses to each symptom, excluding “Other”, and thus ranges from 0 to 90.

Table S6. CarGOQoL Index Values [a]

	Mean (SD)	Median (IQR)
	N = 104	
Psychological well-being	51.56 (23.45)	50.00 (31.25 - 68.75)
Burden	71.94 (22.89)	75.00 (56.25 - 93.75)
Relationship with health care	62.74 (23.68)	66.67 (50.00 - 77.08)
Administration and finances	80.69 (21.86)	91.67 (66.67 - 100.00)
Coping	60.34 (26.72)	58.33 (41.67 - 83.33)
Physical well-being	61.78 (22.96)	62.50 (43.75 - 81.25)
Self-esteem	72.60 (24.14)	75.00 (62.50 - 100.00)
Leisure time	57.33 (22.01)	56.25 (37.50 - 75.00)
Social support	56.85 (25.11)	62.50 (37.50 - 75.00)
Private life	45.55 (22.03)	63.79 (53.02 - 74.14)

Abbreviation: **CarGOQoL** = CareGiver Oncology Quality of Life questionnaire; **IQR** = Interquartile Range (25th–75th percentile); **QoL** = Quality of life; **SD** = Standard deviation.

[a] Scores range from 0 to 100. Higher scores indicate a better QoL.

Figure S2. Scatter Plots for EORTC QLQ-C30 Global Health Status and Patient's Perspective on QoL [a]

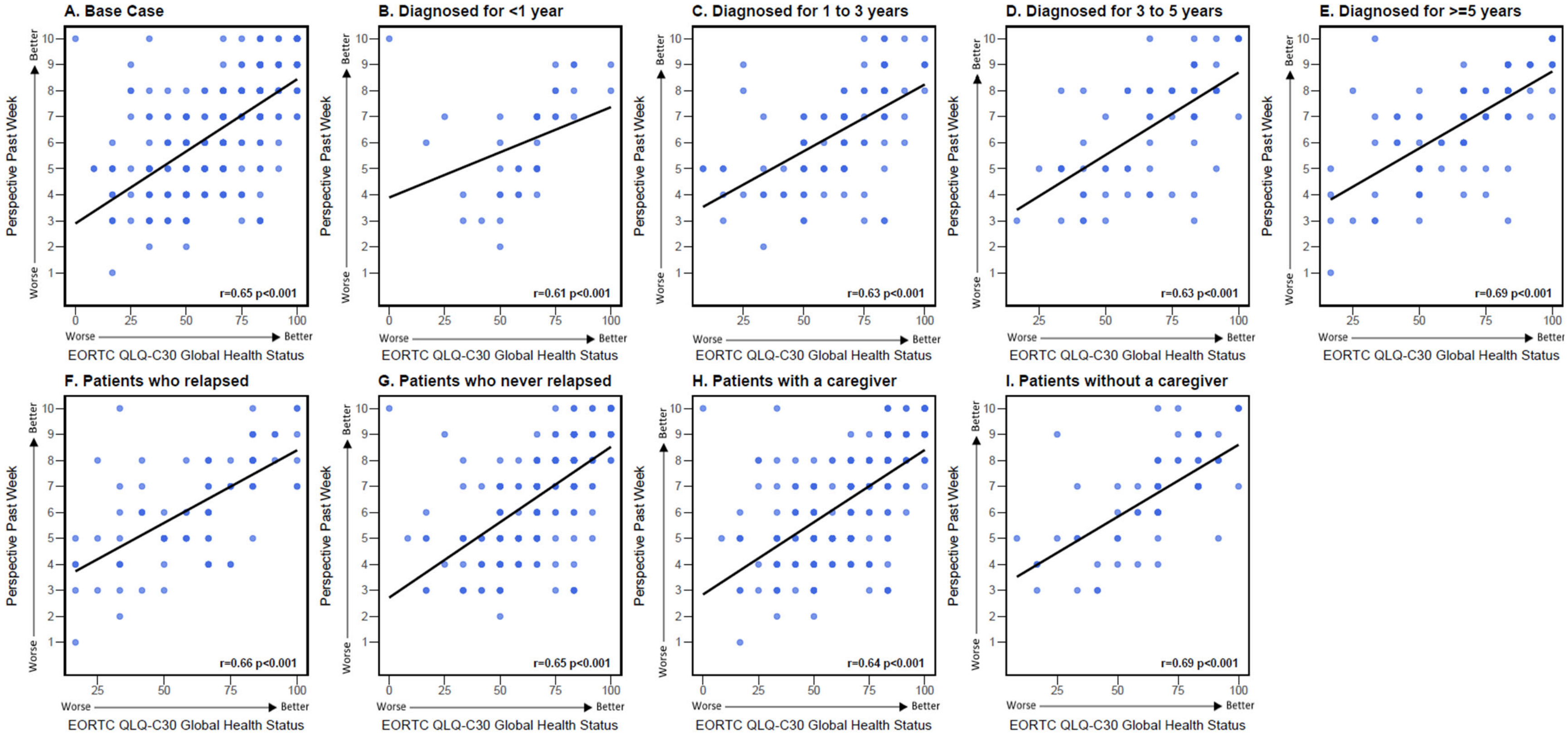

Abbreviations: **EORTC QLQ-C30** = European Organisation for Research and Treatment of Cancer Quality of Life questionnaire core-30 item; **QoL** = Quality of life; **r** = Correlation coefficient.
[a] The correlation coefficient (r) reflects the linear relationship between the score and the patient's ''past week'' perspective, ranging from -1 (perfect inverse correlation) to 1 (perfect positive correlation). A p-value < 0.05 indicates that r is significantly different than zero. No outlier values were identified for EORTC QLQ-C30 Global Health Status and Perspective Past week scores.

Copyright© PeriPharm Inc. Confidential

Figure S3. Scatter Plots for EORTC QLQ-C30 Summary Score and Patient's Perspective on QoL [a]

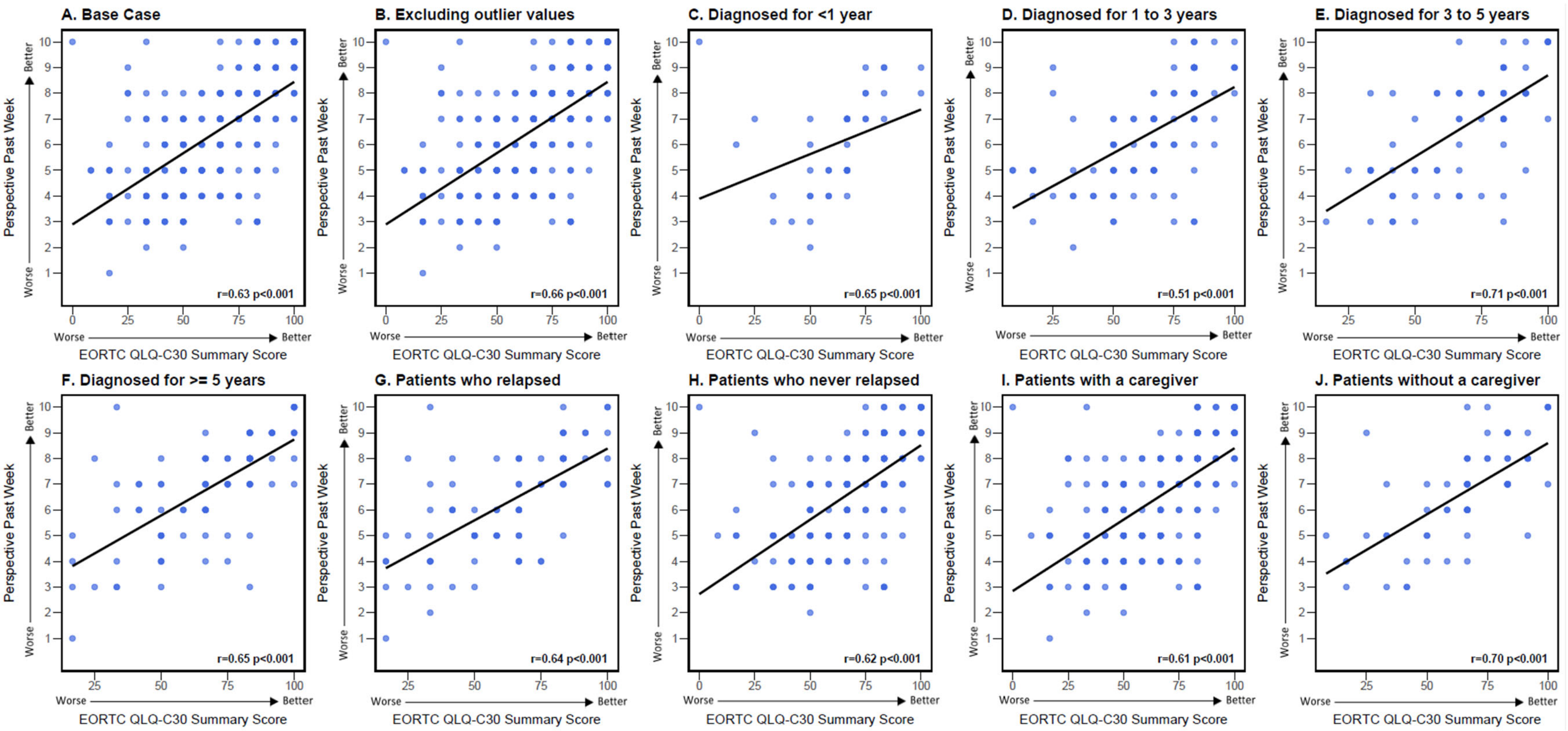

Abbreviations: **EORTC QLQ-C30** = European Organisation for Research and Treatment of Cancer Quality of Life questionnaire core-30 item; **QoL** = Quality of life; **r** = Correlation coefficient.

[a] The correlation coefficient (r) reflects the linear relationship between the score and the patient's ''past week'' perspective, ranging from -1 (perfect inverse correlation) to 1 (perfect positive correlation). A p-value < 0.05 indicates that r is significantly different than zero. No outlier values were identified for Perspective Past week scores and 8 were identified in the EORTC QLQ-C30 Summary scores.

Copyright© PeriPharm Inc.

Figure S4. Scatter Plots for EORTC QLQ-C30 Total Score and Patient's Perspective on QoL [a]

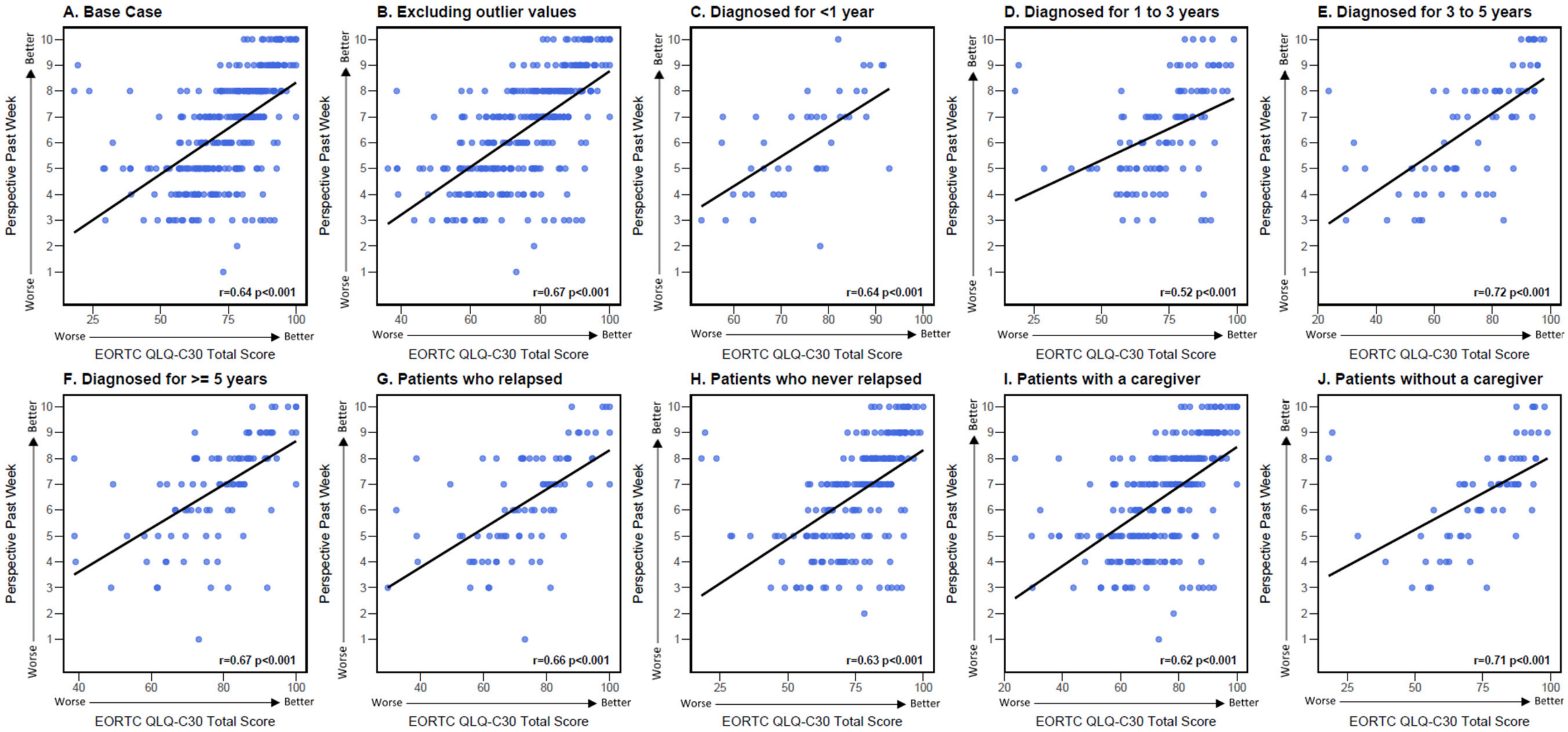

Abbreviations: **EORTC QLQ-C30** = European Organisation for Research and Treatment of Cancer Quality of Life questionnaire core-30 item; **QoL** = Quality of life; **r** = Correlation coefficient.

[a] The correlation coefficient (r) reflects the linear relationship between the score and the patient's ''past week'' perspective, ranging from -1 (perfect inverse correlation) to 1 (perfect positive correlation). A p-value < 0.05 indicates that r is significantly different than zero. No outlier values were identified for Perspective Past week scores and 8 were identified in the EORTC QLQ-C30 Total scores.

Figure S5. Scatter Plots for EORTC QLQ-MY20 Total Score and Patient's Perspective on QoL [a]

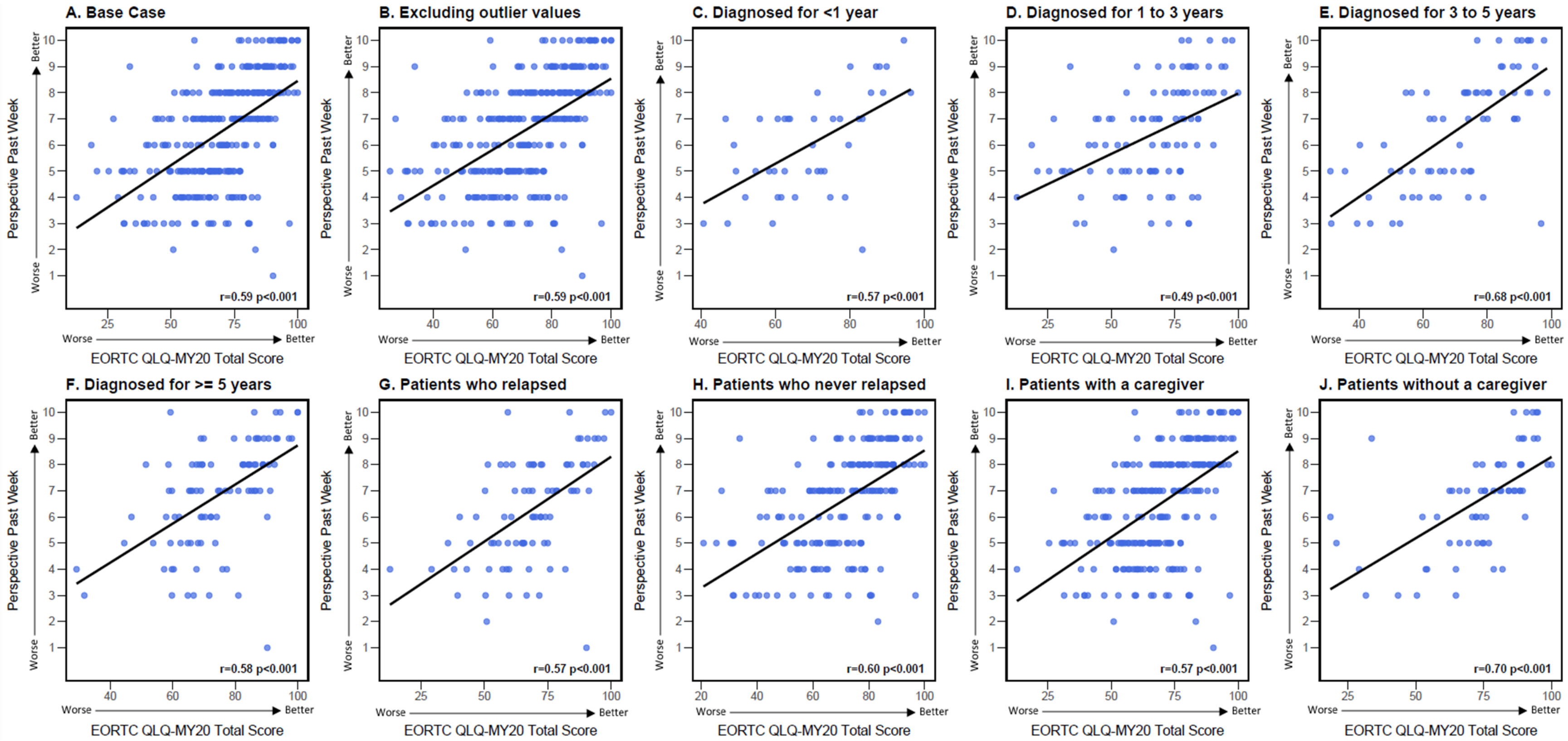

Abbreviations: **EORTC QLQ-MY20** = European Organisation for Research and Treatment of Cancer Quality of Life questionnaire Multiple Myeloma Module; **QoL** = Quality of life; **r** = Correlation coefficient.

[a] The correlation coefficient (r) reflects the linear relationship between the score and the patient's ''past week'' perspective, ranging from -1 (perfect inverse correlation) to 1 (perfect positive correlation). A p-value < 0.05 indicates that r is significantly different than zero. No outlier values were identified for Perspective Past week scores and 3 were identified in the EORTC QLQ-MY20 Total scores.

Figure S6. Scatter Plots for EQ-5D-5L Index Score and Patient's Perspective on QoL [a]

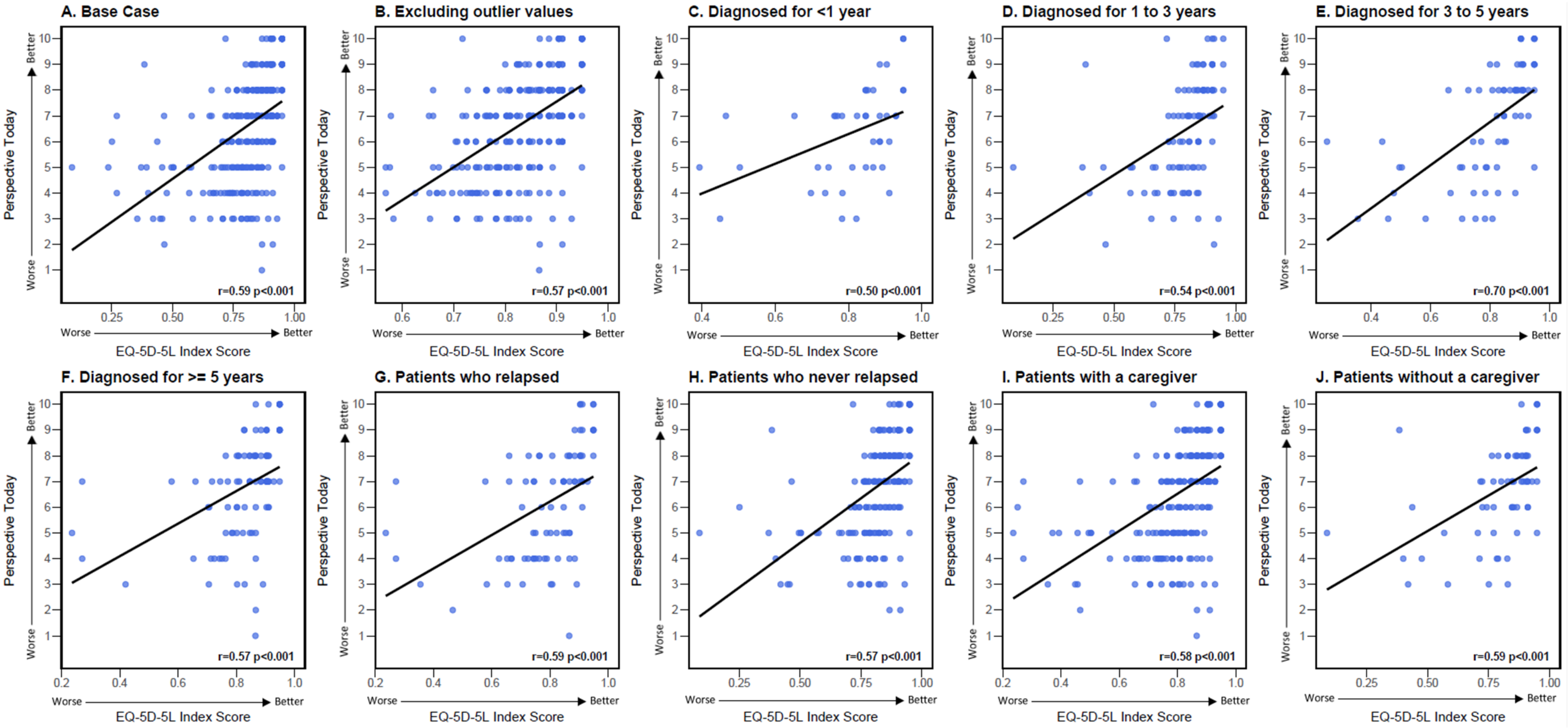

Abbreviations: **EQ-5D-5L** = EuroQol 5 Dimensions 5 Level; **QoL** = Quality of life; **r** = Correlation coefficient.

[a] The correlation coefficient (r) reflects the linear relationship between the score and the patient's perspective ''today'', ranging from -1 (perfect inverse correlation) to 1 (perfect positive correlation). A p-value < 0.05 indicates that r is significantly different than zero. No outlier values were identified for Perspective Today scores and 21 were identified in the EQ-5D-5L index scores.

Figure S7. Scatter Plots for ESAS-R Total Distress Score and Patient's Perspective on QoL [a]

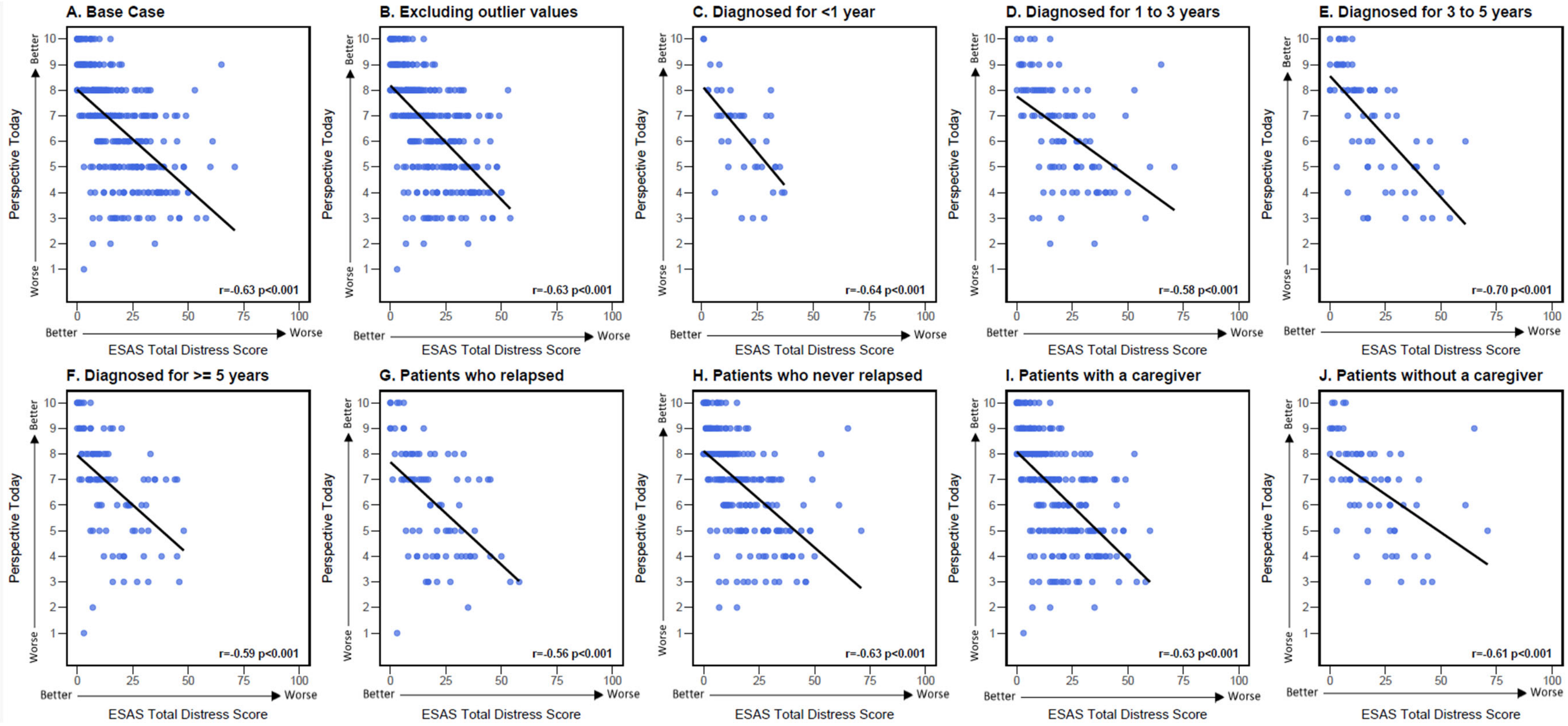

Abbreviations: **ESAS-R** = Edmonton Symptoms Assessment Scale – Revised; **QoL** = Quality of life; **r** = Correlation coefficient.

[a] The correlation coefficient (r) reflects the linear relationship between the score and the patient's perspective ''today'', ranging from -1 (perfect inverse correlation) to 1 (perfect positive correlation). A p-value < 0.05 indicates that r is significantly different than zero. No outlier values were identified for Perspective Today scores and 5 were identified in the ESAS-R Total Distress scores.

Figure S8. Scatter Plots for CarGOQoL Index Score and Caregiver's Perspective on QoL [a]

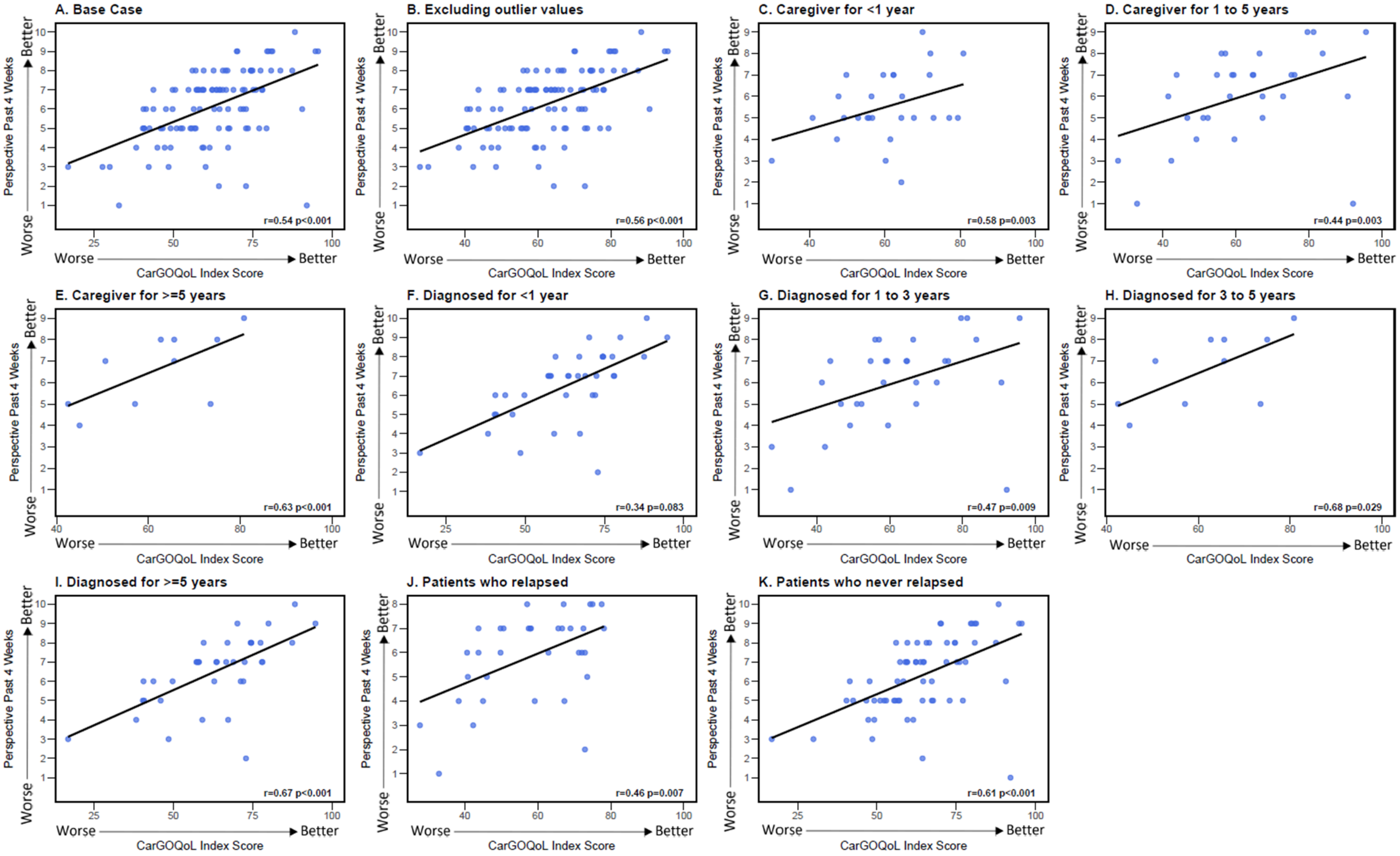

Abbreviations: **CarGOQoL** = CareGiver Oncology Quality of Life questionnaire; **QoL** = Quality of life; **r** = Correlation coefficient.

[a] The correlation coefficient (r) reflects the linear relationship between the score and the patient's ''past 4 weeks'' perspective, ranging from -1 (perfect inverse correlation) to 1 (perfect positive correlation). A p-value < 0.05 indicates that r is significantly different than zero. 2 outlier values were identified for Perspective Past 4 weeks scores and 1 in the CarGOQoL Index scores.

Table S7. Questionnaire correlation with the patient's perspective – Subgroup analysis by type of insurance

Type of medical insurance	Questionnaire correlation with the patient's perspective*, r (95% CI); p-value			
	QLQ-C30 Total score	QLQ-MY20 Total score	EQ-5D-5L Index score	ESAS-R Total distress score
Private (N=127)	0.59 (0.46 to 0.70); <0.01	0.52 (0.38 to 0.64); <0.01	0.51 (0.37 to 0.63); <0.01	-0.58 (-0.69 to -0.45); <0.01
Public (N=75)	0.62 (0.45 to 0.75); <0.01	0.68 (0.53 to 0.79); <0.01	0.55 (0.37 to 0.70); <0.01	-0.62 (-0.74 to -0.45); <0.01
Private and public (N=95)	0.73 (0.62 to 0.82); <0.01	0.62 (0.47 to 0.73); <0.01	0.70 (0.57 to 0.79); <0.01	-0.69 (-0.78 to -0.56); <0.01

* Patient's perspective with the recall of the past week for QLQ-C30 Total score and QLQ-MY20 Total score, and with the recall of today for EQ-5D-5L Index score and ESAS-R Total distress score.
